# *In Vivo* Targeting of Escherichia coli with Vancomycin-Arginine

**DOI:** 10.1128/AAC.02416-20

**Published:** 2021-03-18

**Authors:** Lewis F. Neville, Itamar Shalit, Peter A. Warn, Marc H. Scheetz, Jiuzhi Sun, Madeline B. Chosy, Paul A. Wender, Lynette Cegelski, Jacob T. Rendell

**Affiliations:** a SuperTrans Medical Ltd., Ness Ziona, Israel; b Magic Bullet Consulting, London, United Kingdom; c Department of Pharmacy Practice, Chicago College of Pharmacy and Department of Pharmacology, College of Graduate Studies, Midwestern University, Downers Grove, Illinois, USA; d Department of Chemistry, Stanford University, Stanford, California, USA; e Department of Chemical and Systems Biology, Stanford University, Stanford, California, USA

**Keywords:** *Escherichia coli*, Gram-negative bacteria, antibiotic resistance, arginine, cationic peptides, multidrug resistance, vancomycin conjugate

## Abstract

The ability of vancomycin-arginine (V-r) to extend the spectrum of activity of glycopeptides to Gram-negative bacteria was investigated. Its MIC towards Escherichia coli, including β-lactamase expressing Ambler classes A, B, and D, was 8 to 16 μg/ml. Addition of 8 times the MIC of V-r to E. coli was acutely bactericidal and associated with a low frequency of resistance (<2.32 × 10^−10^). *In vivo*, V-r markedly reduced E. coli burden by >7 log_10_ CFU/g in a thigh muscle model. These data warrant further development of V-r in combatting E. coli, including resistant forms.

## Abstract (precis)

The ability of vancomycin-arginine (V-r) to extend the spectrum of activity of glycopeptides to Gram-negative bacteria was investigated. Its MIC toward Escherichia coli, including β-lactamase expressing Ambler classes A, B, and D, was 8 to 16 μg/ml.

## INTRODUCTION

**FIG 1:**
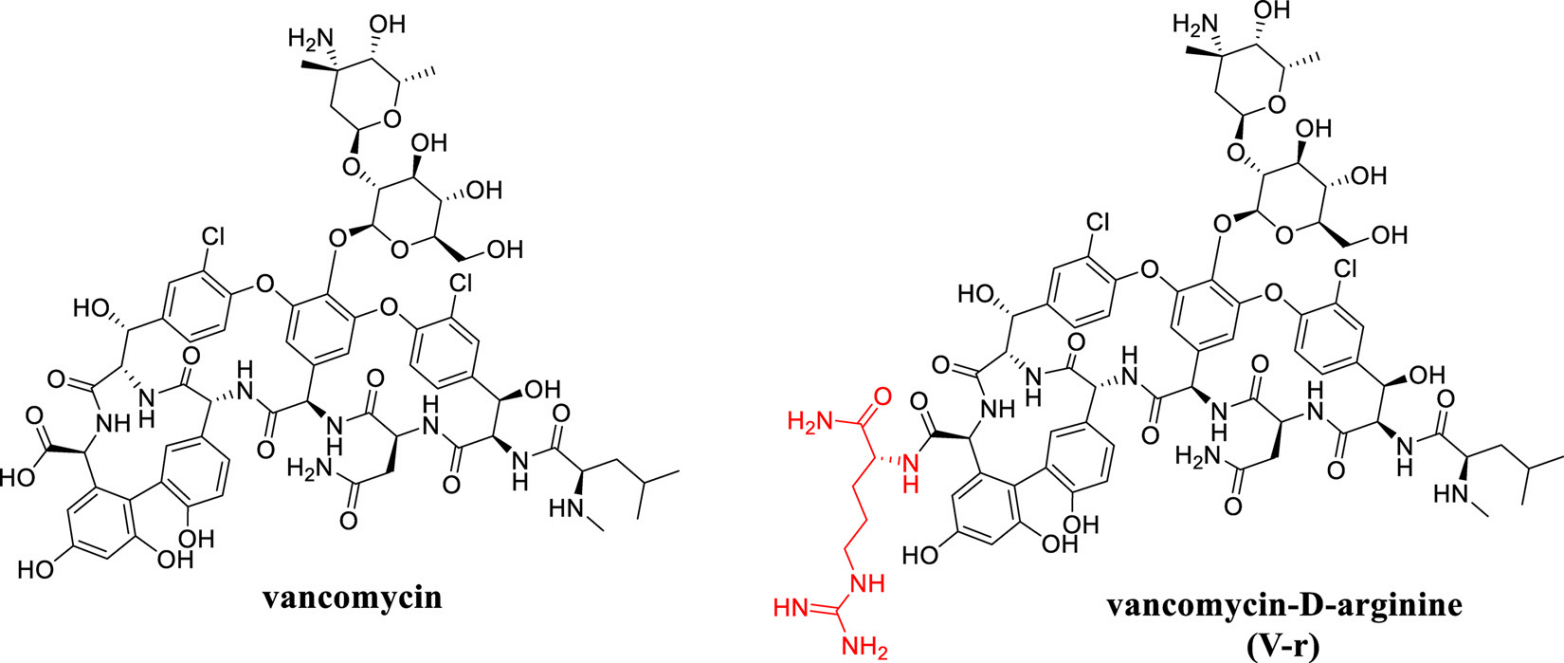
Vancomycin and vancomycin-d-arginine (V-r).

Novel antibiotics are desperately needed to combat priority 1 or urgent-threat pathogens (1–3). With only four new classes of antibiotics introduced into the market since the early 1960s (4), structural modifications of current antibiotics provide an attractive and possibly speedier approach to fulfill this significant unmet clinical need. Vancomycin is a standard-of-care glycopeptide antibiotic for the treatment of Gram-positive infections (5). Numerous reports have demonstrated augmentation of its antimicrobial activity against resistant strains via different chemical modifications (6–9). Furthermore, its molecular structure has been successfully manipulated to create a broader spectrum of activity in the targeting of Gram-negative bacteria via adjuvant, formulation, and cationic/lipophilic interventions (10, 11) or synergy with existing Gram-negative antibiotics (12, 13). Recently, the covalent conjugation of l-arginine to vancomycin, to produce vancomycin-l-arginine (V-R), led to promising Gram-negative properties via a cell wall mode of action (14). These findings encouraged us to further characterize the corresponding diastereomer vancomycin-d-arginine (V-r) in animal models of E. coli infection using the d-isomer of arginine to reduce the risk of conjugate hydrolysis (Fig. 1).

V-r was synthesized in a single chemical step from commercially available vancomycin HCl (StruChem, Wujiang City, China) and d-arginine amide dihydrochloride (Aladdin Chemical Co., Shanghai, China). The crude compound was purified and isolated as the corresponding HCl salt at 95% purity by high-performance liquid chromatography based on a previously described procedure (14). Identity was confirmed by ^1^H nuclear magnetic resonance and time of flight mass spectrometry, and HCl content was quantified by ion-exchange chromatography. In various physicochemical screens, V-r behaved similarly to vancomycin, including no observed cellular cytotoxicity at concentrations ranging from 100 to 750 μM on human erythrocytes, HepG2, and primary renal proximal tubule epithelial cells employing fetal bovine serum-deficient media to negate compound quenching (15) (Table 1).

**TABLE 1:** Physicochemical properties of vancomycin-arginine (V-r) and vancomycin

Physicochemical properties^*a*^	V-r	Vancomycin
Mol wt (free base)	1,604	1,449
LogD (octanol/buffer)	Less than −4.01	−5.14^*b*^
TD solubility in saline (mg/ml)	373	> 50
PPB (mouse/human % bound)	65/76	50/50
Red blood cell lysis (CC_50_, μM)	>750	>750
HepG2 cell cytotoxicity (CC_50_, μM)	>750	>750
hRPTEC biomarkers^*c*^ (CC_50_, μM)	>100	>100
FoR (at 8× MIC)	<2.32 × 10^−10^	Not determined

a TD, thermodynamic; PPB, plasma protein binding; hRPTEC, human renal proximal tubular epithelial cells; CC_50_, concentration at which 50% cytotoxicity is observed; FoR, frequency of resistance.

b LogD vancomycin reported according to Dave and Morris (29).

c Includes cell count, nuclear size, DNA structure, mitochondrial mass, mitochondrial membrane potential, phospholipidosis, and glutathione content.

**FIG 2:**
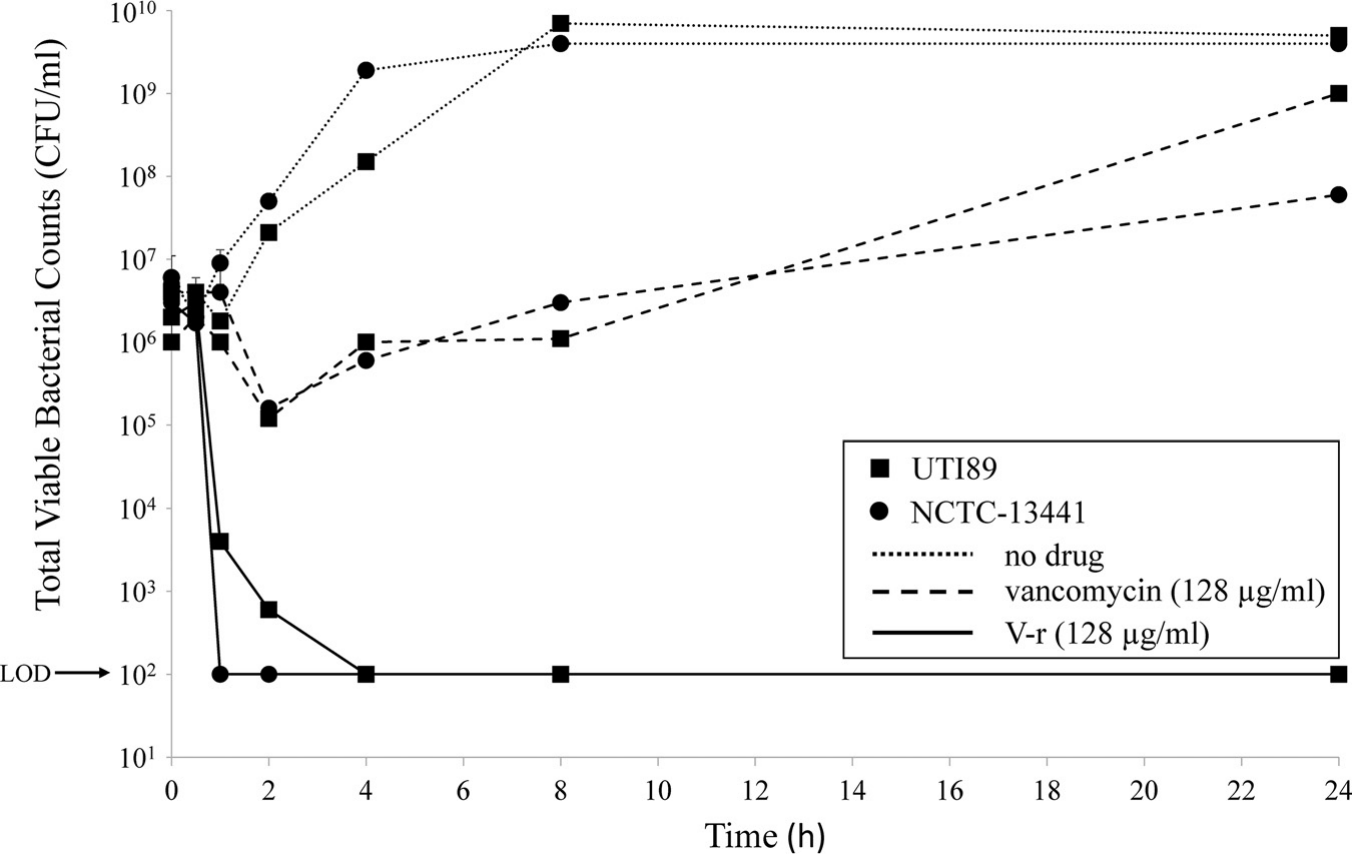
Time-kill of vancomycin-arginine (V-r) and vancomycin against E. coli uropathogens UTI89 and NCTC 13441.

MICs were determined in alignment with CLSI guidelines as previously described for V-R and cationic antimicrobial peptides (14, 16). The MIC range of V-r against 29 different E. coli strains was 8 to 16 μg/ml (MIC_90_, 16 μg/ml), including those with multiple resistance mechanisms (Table 2). The MIC of V-r against the efflux pump mutant strain JW0451-2 was 8 μg/ml, suggesting that V-r is unlikely to be a substrate for efflux in this pathogen. Notably, the MIC of V-r was also 8 μg/ml against two out of five of the Acinetobacter baumannii strains tested. In comparison, the MICs of vancomycin were significantly higher, at 64 to 256 μg/ml, against all E. coli and A. baumannii strains tested. Importantly, the antimicrobial potency of V-r towards a number of Gram-positive bacteria remained intact (Table 2). In frequency-of-resistance (FoR) assays at 8 times the MIC of V-r (128 μg/ml), E. coli ATCC 25922 demonstrated an extremely low FoR, at <2.32 × 10^−10^, which is similar to or lower than those with standard-of-care therapies, such as ciprofloxacin (17, 18). Time-kill assays were performed against uropathogenic E. coli strains, including the sequence type 131 (ST131) NCTC 13341 isolate. V-r, but not vancomycin, demonstrated rapid bactericidal activity to limits of detection (i.e., 100 CFU/ml) within 1 or 4 h of exposure, and this was maintained up to 24 h (Fig. 2).

**TABLE 2:** Antimicrobial susceptibility profiles of V-r and vancomycin

Organism	Strain	Source, resistance mechanism or genotype^*a*^	Ambler class	MIC (μg/ml) of:	
				V-r	Vancomycin
E. coli	ATCC 25922	CLSI susceptible reference strain		16	128
E. coli	UTI89	Clinical isolate from patient with acute bladder infection		16	128
E. coli	NCTC 13441	Uropathogenic E. coli ST131, *bla*_CTX-M-15_, *bla*_OXA-1_, *bla*_TEM-1_, *aac6'-lb-cr*, *mph*(A), *catB4*, *tet*(A), *dfrA7*, *aadA5*, *sul1*	A, D	16	128
E. coli	NCTC 13462	*bla*_CTX-M-2_	A	16	128
E. coli	NCTC 13846	Clinical isolate, bacteremia, UK 2013, EUCAST reference isolate, *mcr-1*		8	64
E. coli	AR055	*bla*_NDM-1_, *mph*(A), *bla*_CMY-6_, *dfrA17*, *sul1*, *tet*(A), *rmtC*, *aac(3)-IIa*, *bla*_OXA-1_, *aadA5*	B, C, D	16	128
E. coli	AR089	*strB*, *bla*_CMY-2_, *tet*(B), *strA*, *sul2*	C	16	128
E. coli	AR0114	*strB*, *bla*_TEM-1B_, *bla*_KPC-3_, *aadB*, *dfrA5*, *sul1*, *strA*, *sul2*, *cmIA1*	A	16	256
E. coli	AR0137	*bla*_NDM-6_, *bla*_OXA-9_, *mph*(A), *bla*_TEM-1A_, *bla*_CMY-42_, *bla*_CTX-M-15_, *dfrA17*, *qnrS1*, *sul1*, *tet*(B), *aadA1*, *aac(3)-IIa*, *bla*_OXA-1_, *aadA5*	B	16	128
E. coli	AR0150	*bla*_NDM-5_, *mph*(A), *bla*_TEM-1B_, *bla*_CMY-42_, *dfrA17*, *sul1*, *tet*(A), *aadA5*	A, B, C	8	128
E. coli	AR0346	*mcr-1*, ESBL	A	16	256
E. coli	AR0349	*mcr-1*, ESBL	A	16	128
E. coli	AR0350	*mcr-1*	-	16	128
E. coli	AR0493	*mcr-1*, ESBL	A	16	256
E. coli	AR0494	*mcr-1*	-	8	128
E. coli	B096a	Clinical isolate (UK) 2016, AmpC	C	16	128
E. coli	B808	Clinical isolate (UK) 2016, *bla*_TEM-1_, *bla*_CTX-M-15_	A	16	256
E. coli	ATCC BAA-2340	*bla*_KPC_	A	16	128
E. coli	ATCC BAA-2469	*bla*_NDM-1_	B	16	128
E. coli	ExPEC H5	Clinical isolate (UK)		8	128
E. coli	H4/5	Clinical isolate, *bla*_TEM-1_, *bla*_CTX-M-15_	A	16	256
E. coli	IR3	Clinical isolate, *bla*_NDM-1_	B	8	128
E. coli	IR45	Clinical isolate, *bla*_NDM-1_	B	16	128
E. coli	IR57	Clinical isolate, *bla*_NDM-1_	B	16	256
E. coli	Swiss 2 (AF45)	Clinical isolate (South Africa) ST101, *mcr-1*		16	128
E. coli	Swiss 13	Clinical isolate (France) ST69, *mcr-1*		16	128
E. coli	Swiss 15	Clinical isolate (Switzerland) ST446, *mcr-1*, *bla*_CTX-M_	A	16	128
E. coli	BW25113	Parent strain of BW25113Δ*acrB::kan* mutant		8	128
E. coli	JW0451-2	BW25113Δ*acrB::kan,* AcrB-deficient mutant, defective in ArcAB-TolC multidrug efflux system		8	128
A. baumannii	ATCC 19606	Isolated from urine, genome-sequenced strain		32	128
A. baumannii	ACC00527	Clinical respiratory isolate (USA) 2012, *bla*_OXA-24_	D	8	128
A. baumannii	B803	Clinical isolate (UK) 2016		32	128
A. baumannii	GS2AB1	Multiresistant clinical isolate (southern Europe) 2017		16	128
A. baumannii	Naval-81	Clinical isolate (USA) 2006		8	128
S. aureus	ATCC 29213	CLSI susceptible reference strain		2	2
S. aureus	NRS 384	USA300-0114 MRSA, community associated		0.5	2
E. faecalis	ATCC 29212	CLSI QC strain		1	2
E. faecalis	B575	Clinical isolate (northwest UK)		1	2
S. agalactiae	B057	Clinical isolate (northwest UK)		0.06	0.5
S. agalactiae	B063	Clinical isolate (northwest UK)		0.06	1
S. pneumoniae	ATCC 49619	Reference strain		0.25	0.5
S. pneumoniae	3259-03	Clinical isolate (northwest UK)		0.5	0.5

a ESBL, extended-spectrum β-lactamase.

Plasma pharmacokinetics (PK) of V-r after subcutaneous (s.c.) administration (20 and 121 mg/kg) was determined in naive male CD-1 mice (*n* = 3/group) using liquid chromatography-tandem mass spectrometry for analysis with a lower limit of quantitation of 5 ng/ml (Table 3). V-r displayed first-order elimination, similar to vancomycin, after s.c. administration (19, 20). Prior to efficacy studies, a single s.c. administration of V-r was shown to be well tolerated in male CD-1 mice (*n* = 3) at the highest dose tested (800 mg/kg).

**TABLE 3:** PK parameters of V-r in CD-1 mice after s.c. administration

PK parameter^*a*^	V-r at 20 mg/kg	V-r at 121 mg/kg
Half-life (h)	0.87	1.29
*C*_max_ (mg/liter)	20.4	98.4
Clearance (ml/min/kg)	7.8	5.4
AUC (mg · h/liter)	42.7	366
V_d_ (liter/kg)	0.59	0.60

a *C*_max_, maximum concentration of drug in plasma; AUC, area under the curve; V_d_, volume of distribution.

**FIG 3:**
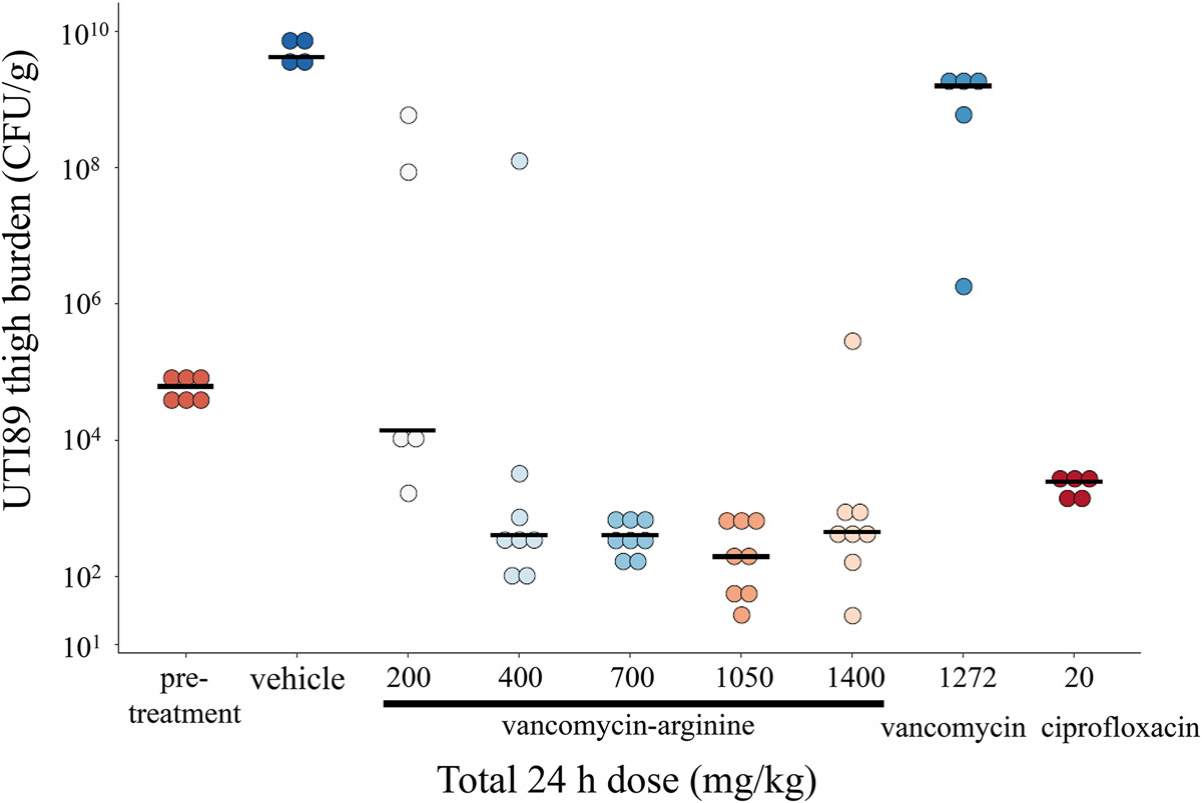
Efficacy of V-r in reducing E. coli UTI89 burden in a 24-h thigh muscle infection model in neutropenic CD-1 mice.

Using a screening-based strategy, preliminary proof-of-concept studies with V-r employed an abbreviated 9-h thigh muscle infection model in male CD-1 mice rendered neutropenic (21). To that end, an E. coli ATCC 25922 isolate was inoculated at 9.7 × 10^4^ CFU into both thigh muscles per mouse (*n* = 5 per experimental group). V-r was administered s.c. every 2 h (110 to 880 mg/kg total dose) starting 1 h postinfection. At 9 h, thigh homogenates were prepared, and CFU were enumerated after culture on CLED (cystine-, lactose-, and electrolyte-deficient) agar. Compared to pretreatment and vehicle burdens of 5.1 ± 0.2 and 7.1 ± 0.1 log_10_ CFU/g tissue, respectively, V-r exhibited a dose-dependent reduction in bacterial burden of 1.2 to 3.4 log_10_ compared with vehicle (Kruskal-Wallis one-way analysis of variance using StatsDirect Statistical Analysis Software) (Table 4). V-r doses at 440 and 880 mg/kg afforded 1.0- and 1.3-log_10_ reductions below stasis, respectively, with an extrapolated static dose of 215 mg/kg. As anticipated, vancomycin failed to significantly impact E. coli burden at a dose equivalent to the highest dose of V-r. In a 24-h thigh muscle infection model, E. coli UTI89 was inoculated at 7.8 × 10^4^ CFU into one thigh muscle per mouse (*n* = 5 to 8 per group) and treated with V-r (total dose, 200 to 1,400 mg) using an every-6-h dosing regimen from 1 h postinfection. All doses of >200 mg/kg significantly reduced burden below stasis by up to 2.7 log_10_ CFU/g. These bactericidal effects of V-r were statistically superior to those of ciprofloxacin, which induced a 1.4 log_10_ reduction from stasis (Fig. 3 and Table 5). Overall, V-r caused an ∼4 to 7.5 log_10_ reduction in bacterial burden, compared with vehicle control, over the entire dose range.

**TABLE 4:** Efficacy of V-r in an E. coli ATCC 25922 thigh muscle infection model (9 h) in neutropenic CD-1 mice

Group, total dose over 9 h (mg/kg)	Log_10_ (group geometric mean ± SD CFU/g)	Log_10_ change from vehicle (CFU/g)	*P* value (versus vehicle)
Pretreatment	5.1 ± 0.18	−2.01	0.0045
Vehicle	7.11 ± 0.12	0	0
V-r, 110	5.87 ± 0.60	−1.24	0.0415
V-r, 440	4.14 ± 0.63	−2.97	<0.0001
V-r, 880	3.76 ± 0.40	−3.35	<0.0001
Vancomycin, 800	6.60 ± 0.66	−0.51	Not significant

**TABLE 5:** Efficacy of V-r in reducing E. coli UTI89 burden in 24-h thigh muscle infection model in neutropenic CD-1 mice

Group, total dose over 24 h (mg/kg)	Log_10_ (group geometric mean ± SD CFU/g)	Log_10_ change from vehicle (CFU/g)	*P* value (versus vehicle)
Pretreatment	4.76 ± 0.18	−4.95	0.0248
Vehicle	9.71 ± 0.17	0	0
V-r, 200	5.60 ± 2.28	−4.11	0.0217
V-r, 400	3.27 ± 1.88	−6.43	<0.0001
V-r, 700	2.58 ± 0.25	−7.13	<0.0001
V-r, 1,050	2.08 ± 0.89	−7.63	<0.0001
V-r, 1,400	2.68 ± 1.38	−7.03	<0.0001
Vancomycin, 1,272	8.48 ± 1.31	−1.23	Not significant
Ciprofloxacin, 20	3.32 ± 0.14	−6.39	<0.0007

The MIC data confirm previous findings that the coupling of arginine with vancomycin bestows significant antimicrobial activity of the V-r conjugate against E. coli infection while remaining effective against methicillin-resistant Staphylococcus aureus (MRSA) (14). Such *in vitro* findings were effectively translated into thigh muscle infection models, where a total 24-h dose of 250 mg/kg V-r reduced E. coli burden to pretreatment (stasis) levels. Since area under the curve over 24 h in the steady state divided by the MIC (AUC/MIC ratio) is the primary PK/pharmacodynamic predictor of vancomycin (5), this static dose corresponds to a total AUC/MIC of 47.3. Based on a free (*f*) fraction of 35%, as determined in plasma protein binding studies (Table 1), the *f*AUC/MIC of V-r was 16.5. As an approximation of exposure using allometric scaling (22), this would be equivalent to a human dose of ∼20 mg/kg, with a dose of 28 mg/kg required to elicit an additional 1-log_10_ kill. Such allometric doses of V-r are in line with the daily and loading doses of vancomycin in humans (5).

The positive efficacy data support the notion that the cationic feature of arginine within V-r allows for breaching of the stubborn outer membrane of E. coli isolates and possibly other Gram-negative bacteria (14). The sequelae of events leading to V-r-mediated E. coli eradication likely involve (i) improved cell surface association with negatively charged groups, (ii) effective translocation across the outer membrane leading to enhanced drug uptake, and (iii) disruption of peptidoglycan synthesis within the periplasmic space (6, 14). To our knowledge, the current findings describe the first report of a marked abrogation of E. coli burden *in vivo* with a minimally modified vancomycin-cationic transporter conjugate. Previously, it was reported that vancomycin-QC14, a strongly lipophilic/cationic molecule, reduced thigh muscle infection of a carbapenem-resistant A. baumannii strain (23). Because V-r was highly effective in time-kill assays against E. coli NCTC 13441, a pandemic uropathogenic clone (24), a logical next step would be to evaluate the conjugate in a model of urinary tract infection (UTI). Based on the high renal elimination of vancomycin in humans (25) in a nonmetabolized form (26), it is reasonable to hypothesize that V-r may drive a highly targeted therapeutic intervention to combat E. coli-associated UTIs.

These data further underscore a precedent for creating a novel Gram-negative active agent by transforming a commonly used and selective Gram-positive antibiotic by introducing certain cationic features through a simple and scalable synthesis protocol (14). Such an approach, in consort with effective *in silico* predictions (27, 28), might expedite antibiotic development and increase the overall probability of success of drug candidates. Most important, this would help to arrest the insidious pandemic of difficult-to-treat bacterial infections.
